# Invisible youth during times of Covid

**DOI:** 10.1192/bjb.2021.20

**Published:** 2021-04

**Authors:** Melissa Beaumont, Kate Chalker, Layla Clayton, Emily Curtis, Heidi Hales, Duncan Harding, Rhiannon Lewis, Gabrielle Pendlebury

**Affiliations:** Team Lead & Clinical Nurse Specialist, Health & Wellbeing Team Feltham YOI, Central and North West London NHS Trust, UK; Wye CAMHS Clinical Nurse Specialist Hounslow YOT West London NHS Trust, UK; Assistant Psychologist, Health & Wellbeing Team Feltham YOI, Central and North West London NHS Trust, UK; Youth Justice Liaison and Diversion Hammersmith and Fulham Youth Offending Service, UK; Consultant Psychiatrist, North West London FCAMHS, West London NHS Trust, UK; Consultant Psychiatrist, South London FCAMHS, UK; Consultant Clinical Psychologist and Clinical Lead for SECURE STAIRS and the Enhanced Support Unit, HMYOI Feltham, Central and North West London NHS Trust, UK; Consultant Adolescent Psychiatrist and Medico-legal Adviser, FCAMHS at The Port, Tavistock and Portman NHS Trust, UK. Email: g_pendlebury@hotmail.com, @PendlesGabriel

Covid-19 continues to devastate, the elderly and those in care homes being particularly vulnerable. However, there is an unexpected population that is at great risk of morbidity due to Covid-19, the adolescent forensic population. This increased morbidity is a result of the care offered by statutory agencies being greatly diminished across all settings owing to the pandemic.

One of the first things that you learn in adolescent forensic psychiatry is that perpetrators are also victims, and it can be hard to distinguish between the two. This does not excuse the crimes they have committed but does add an extra complexity to their treatment. Young people who present with complex forensic issues are particularly vulnerable, often having histories that include early trauma, repeated loss, attachment issues, learning difficulties and mental health problems.^1^ This population is notoriously difficult for professionals to engage with, for many of the above reasons but also because of the possible consequences for them and their families of talking about the criminal aspects of their lives. The reduced consistency that services currently provide has affected their engagement and the possibility of a therapeutic alliance, thus increasing risk for themselves and the public.

A further contributing factor to increased morbidity has been school closures and agencies working remotely, leading to reduced access to support and structure, which has exacerbated vulnerabilities. Challenging behaviours were previously mitigated by the provision of education and other prosocial activities; the reduction has led to increased episodes of violence.^2^ This has particularly affected young people with neurodevelopmental disorders and special educational needs, with the effects likely to be long term, complicated by loneliness and a disconnection from their community.

Youth custody has had to be increasingly vigilant to ensure the safety of detainees and prevent Covid transmission. There is a need to isolate those being transferred into custody in the first 2 weeks to prevent transmission of Covid.^3^ It is known that this is the time of greatest risk of suicide for young people in custody, when young people are now needing to isolate for Covid, thus increasing isolation and risk. Staff shortages, education closure and the need for Covid ‘bubbles’ has meant extended time alone in cells even after those first few weeks, which increases the risk of self-harm and suicide.^4^ Furthermore, the pandemic has led to a backlog in the courts, and concern over community services has meant that more young people are being remanded and for longer periods. It is of note that the majority (63%) of children given custodial remand did not subsequently receive a custodial outcome in 2018.^5^ These factors – extra time in cells and longer time on remand – can mean the compounding of an already traumatic experience for many in youth custody.

Young people with a combination of mental health and forensic issues are placed in secure adolescent psychiatric units to receive appropriate treatment. The effect of the Covid pandemic on staffing in these units has negatively affected the availability of support, and things that are often a lifeline for these young people, such as community access, planned leave and family visits, have been cancelled. A bottleneck has occurred, with transition back to the community being stalled owing to the services around the young person not being readily available to facilitate these moves or provide the relevant opportunities in the community.

Isolated young people with social communication, cognitive or emotional difficulties are at increased risk of exploitation by others in all settings. In the community, risks relevant to this group go beyond the risk of offending and risk to others and include being groomed into gang-related activities. Such gangs appear to have been more active and accessible to this group of young people during this time (including county lines and other criminal exploitation).

We realise that everyone is having a difficult time at the moment but believe it is essential to highlight some of the issues that this frequently invisible population is experiencing, in the hope that they will not be forgotten.

However, it is not all doom and gloom; with any change there are gains as well as losses, and the gains have highlighted the dedication of the professionals working with this population. They have stepped up to the mark, through increased productivity, improved multi-agency working, increased uptake of virtual conferencing and appropriate information-sharing, with the aim of keeping these young people safe. The young people have noted these efforts and there has been feedback that this way of working has made them feel safer, so we hope, as a network, that we will be able to maintain the benefits as we return to the new normality.

This letter was compiled by the London Youth Justice Child and Adolescent Mental Health Services (CAMHS) Forum, which comprises professionals working in the following services: Youth Justice Liaison and Diversion, Youth Offending Teams (YOTs), Community Forensic CAMHS (FCAMHS), Youth Offending Institutions (YOIs, including SECURE STAIRS implementation) and secure adolescent units.
